# Comprehensive tissue-specific gene set enrichment analysis and transcription factor analysis of breast cancer by integrating 14 gene expression datasets

**DOI:** 10.18632/oncotarget.14286

**Published:** 2016-12-21

**Authors:** Wen-Xing Li, Kan He, Ling Tang, Shao-Xing Dai, Gong-Hua Li, Wen-Wen Lv, Yi-Cheng Guo, San-Qi An, Guo-Ying Wu, Dahai Liu, Jing-Fei Huang

**Affiliations:** ^1^ Institute of Health Sciences, Anhui University, Hefei 230601, Anhui, China; ^2^ State Key Laboratory of Genetic Resources and Evolution, Kunming Institute of Zoology, Chinese Academy of Sciences, Kunming 650223, Yunnan, China; ^3^ Center for Stem Cell and Translational Medicine, School of Life Sciences, Anhui University, Hefei 230601, Anhui, China; ^4^ Department of Biostatistics, School of Life Sciences, Anhui University, Hefei 230601, Anhui, China; ^5^ Kunming College of Life Science, University of Chinese Academy of Sciences, Kunming 650204, Yunnan, China; ^6^ Hongqiao International Institute of Medicine, Shanghai Tongren Hospital/Faculty of Public Health, Shanghai Jiao Tong University School of Medicine, Shanghai 200025, China; ^7^ KIZ-SU Joint Laboratory of Animal Models and Drug Development, College of Pharmaceutical Sciences, Soochow University, Kunming 650223, Yunnan, China; ^8^ Collaborative Innovation Center for Natural Products and Biological Drugs of Yunnan, Kunming 650223, Yunnan, China; ^9^ Chinese University of Hong Kong Joint Research Center for Bio-resources and Human Disease Mechanisms, Kunming 650223, Yunnan, China

**Keywords:** breast cancer, tissue specific, gene expression, transcription factors, GSEA

## Abstract

Breast cancer is the most commonly diagnosed malignancy in women. Several key genes and pathways have been proven to correlate with breast cancer pathology. This study sought to explore the differences in key transcription factors (TFs), transcriptional regulation networks and dysregulated pathways in different tissues in breast cancer. We employed 14 breast cancer datasets from NCBI-GEO and performed an integrated analysis in three different tissues including breast, blood and saliva. The results showed that there were eight genes (CEBPD, EGR1, EGR2, EGR3, FOS, FOSB, ID1 and NFIL3) down-regulated in breast tissue but up-regulated in blood tissue. Furthermore, we identified several unreported tissue-specific TFs that may contribute to breast cancer, including ATOH8, DMRT2, TBX15 and ZNF367. The dysregulation of these TFs damaged lipid metabolism, development, cell adhesion, proliferation, differentiation and metastasis processes. Among these pathways, the breast tissue showed the most serious impairment and the blood tissue showed a relatively moderate damage, whereas the saliva tissue was almost unaffected. This study could be helpful for future biomarker discovery, drug design, and therapeutic and predictive applications in breast cancers.

## INTRODUCTION

According to the World Health Organization, breast cancer is the most commonly diagnosed cancer in females worldwide. Epidemiology studies have shown that breast cancer incidence has increased by 3.1% per annum between 1980 and 2010 [1]. Based on incidence data from the Globocan 2008 database extrapolated to the projected world population in 2030, the World Economic Forum estimates that nearly 2.2 million new cases of breast cancer will be diagnosed worldwide in 2030 [2]. Furthermore, despite the high treatment success rate, it remains the number one cause of cancer death in women [3]. Approximately 522,000 women worldwide died of breast cancer in 2012, including 324,000 women in less developed countries where the malignancy is currently the leading cause of female cancer deaths, accounting for 14.3% of all cancer fatalities [4].

Several key transcription factors (TFs) play critical roles in the proliferation, invasion and migration of breast cancer cells [5, 6]. A recent study identified 8 TFs that are critical for basal-like breast cancer (BLBC) cell growth, and SOX11 was the only TF required for BLBC growth but not for the growth of non-BLBC cells [7]. PITX2, a paired-like Homeobox transcription factor, contributes to the invasiveness of breast cancer cells, which is an activity that appears to be mediated by the Wnt/beta-Catenin pathway [8]. In addition, another study identified Tbx3 as a novel target of tumor suppressor miR-206 and characterized the miR-206/Tbx3 signaling pathway, which is involved in the proliferation, invasion and maintenance of the cancer stem cell population in breast cancer cells [9].

A cross-tissue gene expression comparison in disease will help us to understand the global molecular landscape and reveal new candidate genes that may serve as suitable drug targets. A recent study reconstructed gene regulatory networks in coronary artery disease from seven tissues (atherosclerotic arterial wall, internal mammary artery, liver, skeletal muscle, visceral fat, subcutaneous fat and whole blood) and identified key drivers including AIP, DRAP1, POLR2I and PQBP1 [10]. Another study revealed several early warning signal genes in liver, muscle and adipose tissues in type 2 diabetes mellitus in rats based on a dynamic network method [11]. Furthermore, a recent clinical study showed that DNA methylation and the gene expression of HIF3A were associated with BMI and insulin resistance by cross-tissue validation (blood, subcutaneous adipose and skeletal muscle) [12].

Several abnormal metabolic pathways, potential biomarkers and drug target genes have already been identified in breast cancer [13–15]. However, to our knowledge, no study has conducted a cross-tissue comparison via the integration of multiple sets of breast cancer gene expression data. Therefore, in the present study, we integrated 14 breast cancer gene expression datasets containing breast, blood and saliva tissues in order to explore the differences in the transcriptional regulation relationships between TFs and TF-target genes as well as impaired pathways in breast cancer and mine the diverse gene signatures among these three tissues.

## RESULTS

### Differentially expressed genes overview

Table 1 shows the details of 14 integrated breast cancer datasets. We mapped 20,307 genes in the integrated breast cancer datasets. Differentially expressed genes in the three subgroups are shown in Table 2. In the breast group, we obtained 1,300 up-regulated and 1,201 down-regulated genes. Furthermore, there were 64 up-regulated and 15 down-regulated genes in the blood group. However, we found no differential expression genes in the saliva group. Commonly and tissue-specific dysregulated genes in the breast and blood group are shown in Supplementary Table 1. We obtained 16 commonly up-regulated genes and 2 commonly down-regulated genes. In addition, 2 genes were up-regulated in the breast and down-regulated in blood. However, 15 genes were down-regulated in the breast but up-regulated in blood. Among these 35 genes, the effect of NCEH1, THOC4, UBE2M, EPB42 or SNORD104 on breast cancer still has yet to be reported.

**Table 1:** Summary of the breast cancer datasets

Series ID	Contributor	Samples^1^	Title	Tissue
GSE8977	Richardson A, 2007	22 (22)	Bone-marrow-derived mesenchymal stem cells promote breast cancer metastasis	Breast
GSE10810	Fárez-Vidal ME, 2008	58 (58)	Gene expression signatures in breast cancer distinguish phenotype charact., histological subtypes, and tumor invasivness	Breast
GSE16391	Haibe-Kains B, 2009	55 (48)	GGI: a potential predictor of relapse for endocrine-treated breast cancer patients in the BIG 1-98 trial	Breast
GSE20266	Zhang L, 2010	20 (20)	Salivary Transcriptomic and Proteomic Biomarkers for Breast Cancer Detection	Saliva
GSE26910	Planche A, 2011	24 (12)	Stromal molecular signatures of breast and prostate cancer	Breast
GSE27562	LaBreche HG, 2011	162 (162)	Expression data from human PBMCs from breast cancer patients and controls	Blood
GSE29431	Lopez FJ, 2011	66 (66)	Identifying breast cancer biomarkers	Breast
GSE31192	Harvell DM, 2011	33 (33)	Molecular Signature of Pregnancy Associated Breast Cancer (PABC)	Breast
GSE35925	Katayama MH, 2012	30 (29)	Calcitriol supplementation effects on Ki67 expression and transcriptional profile of breast cancer specimens from post-menopausal patients	Breast
GSE36765	Willard-Gallo K, 2012	34 (14)	Gene expression profiling of CD4+ T cells infiltrating human breast cancer (Discovery Set)	Blood
GSE42568	Clarke C, 2012	121 (121)	Breast Cancer Gene Expression Analysis	Breast
GSE45827	Gruosso T, 2013	155 (141)	Expression data from Breast cancer subtypes	Breast
GSE50567	Lisowska KM, 2013	41 (41)	BRCA1-related gene signature in breast cancer: the role of ER status and molecular type	Breast
GSE61304	Yenamandra SP, 2014	62 (62)	Novel bio-marker discovery for stratification and prognosis of breast cancer patients	Breast

^1^ All samples of this dataset (samples used in this study).

**Table 2:** Differentially expressed genes in breast cancer

Group	Cases/Controls	Mapped Genes	Up-regulated	Down-regulated
Breast	470/163	20307	1300	1201
Blood	141/35	20307	64	15
Saliva	10/10	20307	0	0

### Tissue-specific dysregulated pathways in breast cancer

**Figure 1:**
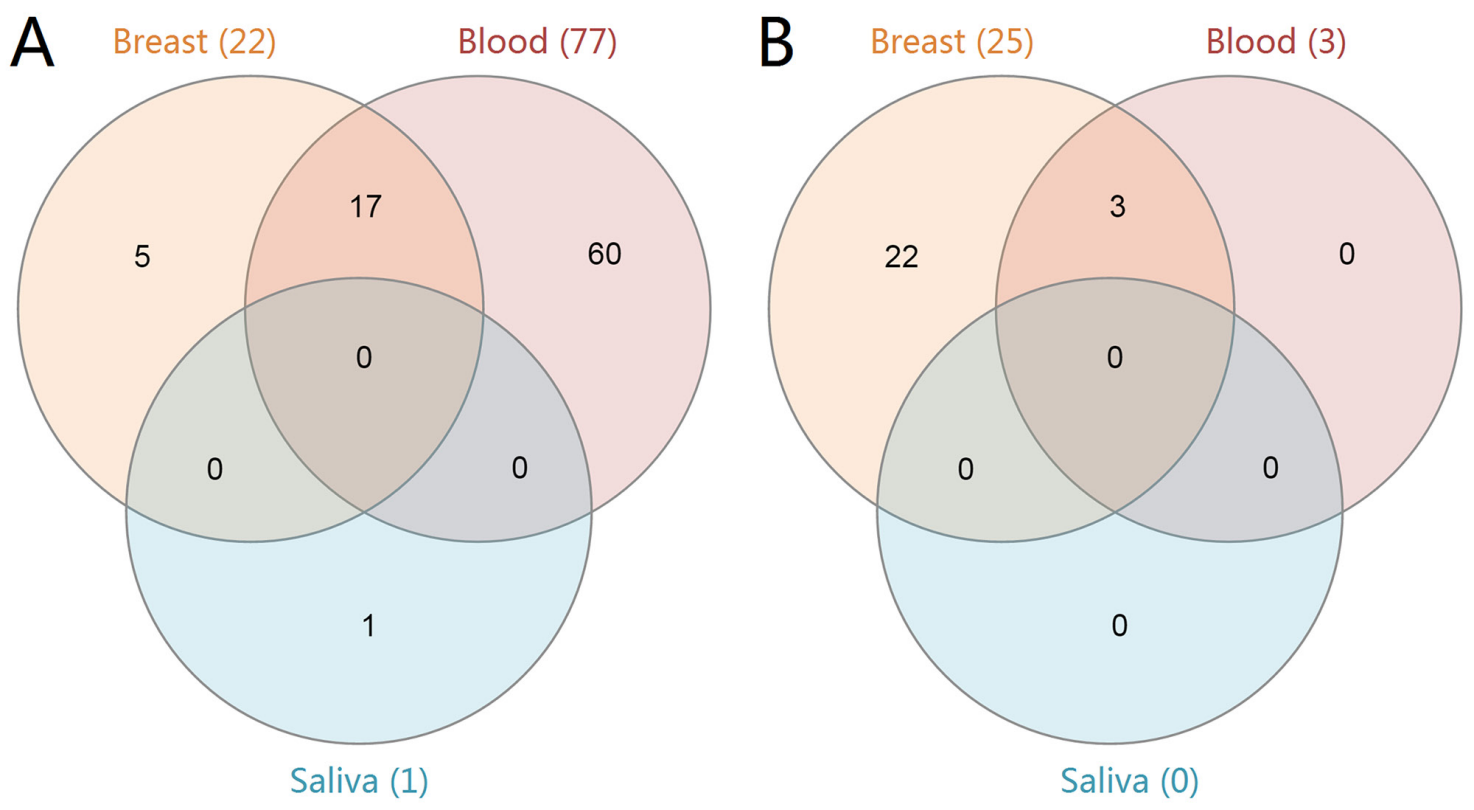
Venn diagram of the enriched KEGG pathways in breast cancer The three groups (breast, blood and saliva) are represented by the orange, red and blue colors, respectively. Panel **A**. shows the up-regulated pathways in each group. Panel **B**. shows the down-regulated pathways in each group.

Gene set enrichment analysis (GSEA) results showed that there were 22 up-regulated and 25 down-regulated pathways in the breast group, and 77 up-regulated and 3 down-regulated pathways in the blood group. Only 1 up-regulated pathway was enriched in the saliva group. The Venn diagram of these enriched pathways is shown in Figure 1. There were 17 commonly up-regulated pathways and 3 commonly down-regulated pathways between breast and blood. Table 3 shows the top 10 significantly enriched pathways in the three groups. The cell cycle, DNA replication, spliceosome, proteasomes, mismatch repair, p53 signaling pathway, nucleotide excision repair and other 10 pathways were up-regulated in the breast and blood groups. Additionally, the down-regulated pathways in the blood group were all enriched in the breast group (olfactory transduction, renin angiotensin system and neuroactive ligand receptor interaction). However, three pathways (fatty acid metabolism, adipocytokine signaling pathway and valine, leucine and isoleucine degradation) were down-regulated in the breast group but up-regulated in the blood group.

**Table 3:** Top 10 dysregulated pathways identified in breast cancer

Group	Up-regulated Pathways	FDR	Down-regulated Pathways	FDR
Breast	Cell cycle	<0.001	Fatty acid metabolism	<0.001
	DMA replication	<0.001	PPAR signaling pathway	<0.001
	Systemic lupus erythematosus	<0.001	Propanoate metabolism	<0.001
	Spliceosome	<0.001	Drug metabolism cytochrome p450	<0.001
	Mismatch repair	<0.001	Adipocytokine signaling pathway	<0.001
	Proteasome	0.001	Retinol metabolism	0.001
	Homologous recombination	0.001	Metabolism of xenobiotics by cytochrome p450	0.001
	Allograft rejection	0.001	Pyruvate metabolism	0.003
	Pyrimidine metabolism	0.002	Butanoate metabolism	0.003
	RNA degradation	0.003	Olfactory transduction	0.014
Blood	Toll-like receptor signaling pathway	<0.001	Olfactory transduction	0.001
	Leishmania infection	<0.001	Neuroactive ligand receptor interaction	0.008
	Ubiquitin mediated proteolysis	<0.001	Renin angiotensin system	0.049
	Cell cycle	<0.001		
	DNA replication	<0.001		
	Acute myeloid leukemia	<0.001		
	NOD-like receptor signaling pathway	<0.001		
	T cell receptor signaling pathway	<0.001		
	Neurotrophin signaling pathway	<0.001		
	Lysosome	<0.001		
Saliva	Ribosome	0.018		

### Expression profiles of TFs and TF-target genes

**Figure 2:**
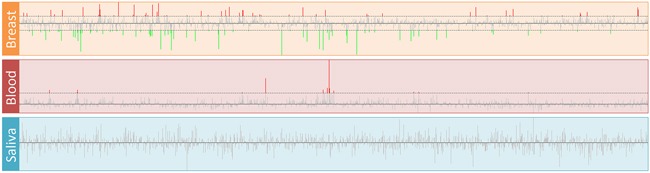
Expression profiles of transcription factors in breast cancer The log2(FC) of all TFs in the breast, blood and saliva groups are displayed. The horizontal dashed lines indicate the cutoff values of log2(FC). The up- and down-regulated TFs are represented by red and green lines, respectively.

**Figure 3:**
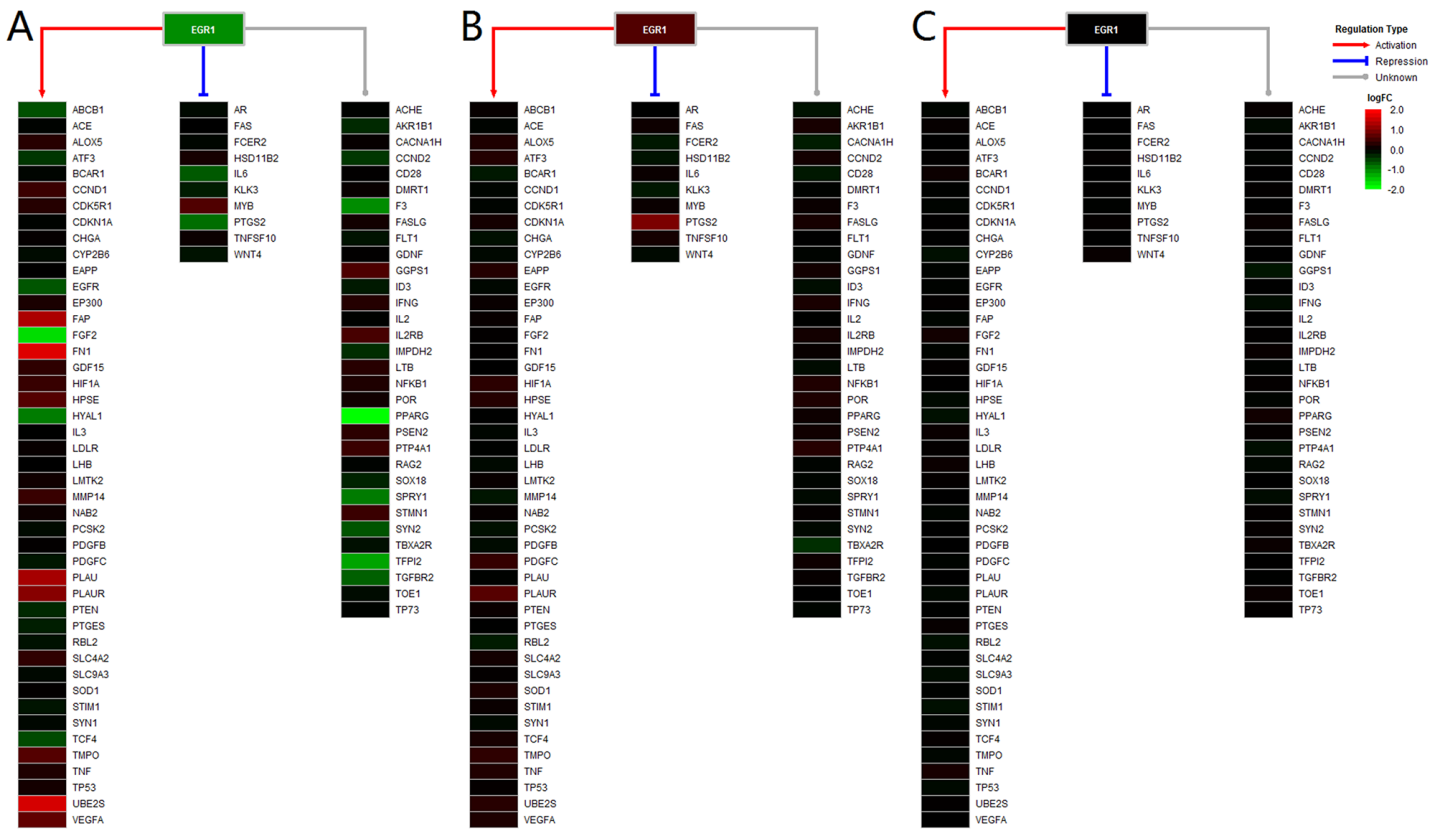
Heatmap of EGR1 and its target genes The gradient color from red to green is expressed as the logFC value of each gene. The red, blue and gray lines show the regulation type of EGR1 on the targets.

**Figure 4:**
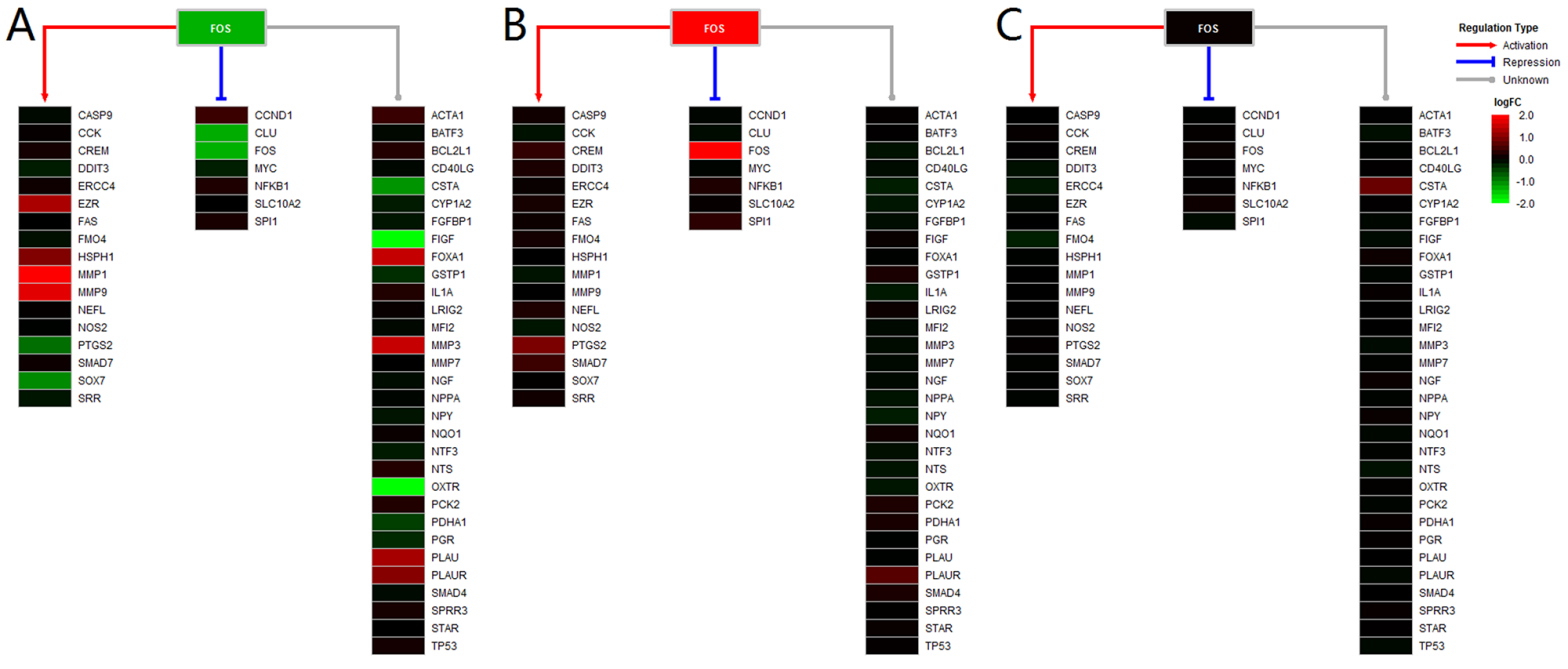
Heatmap of FOS and its target genes The gradient color from red to green is expressed as the logFC value of each gene. The red, blue and gray lines show the regulation type of FOS on the targets.

The expression profiles of 1,469 mapped TFs in three tissues are shown in Figure 2. We obtained 145 and 13 differentially expressed TFs in breast and blood, respectively (Supplementary Table 2). No dysregulated TF was found in the saliva group. There were eight TFs (CEBPD, EGR1, EGR2, EGR3, FOS, FOSB, ID1 and NFIL3) that were down-regulated in the breast group but up-regulated in the blood group. We used TRRUST web server and mapped 11, 87, 5, 3, 55, 3, 1 and 10 target genes of these TFs to our datasets, respectively. Next, we filtered the TFs that have more than 15 target genes and showed their expression profiles. Figure 3 shows the expression profiles of EGR1 and its target genes in the three groups. There were several target genes that were activated by EGR1, such as FAP, FN1, PLAU, PLAUR, UBE2S and VEGFA, in the breast group. Furthermore, we found that PTGS2, PPARG, F3, SPRY1, SYN2, TFPI2 and TGFBR2 were suppressed by EGR1 (Figure 3A). However, these genes were activated by EGR1 or unaffected in the blood group (Figure 3B). No expression change was observed in the saliva group (Figure 3C). The expression profiles of FOS and its target genes are shown in Figure 4. Several target genes were suppressed by FOS in the breast group, such as PTGS2, CLU, FOS, CSTA, FIGF and OXTR (Figure 4A). However, these genes were unaffected or activated by FOS in the blood group (Figure 4B). In the saliva group, we found only CSTA was activated by FOS (Figure 4C).

### Tissue-specific transcriptional regulatory network

The tissue-specific transcriptional regulatory networks (TRNs) of breast, blood and saliva are shown in Supplementary Figure 3-5. The TRN properties of three tissues were listed in Supplementary Table 3. We found that breast TRN had the highest clustering coefficient of 0.083; this value in blood TRN is 0.038 and 0.000 in saliva TRN. Furthermore, breast TRN showed the highest connected component of 153, followed by blood TRN (116) and saliva TRN (28). In addition, breast TRN had the most multi-edge node pairs of 95, followed by blood TRN (55) and saliva TRN (9). The betweenness centrality of three TRNs were displayed in Supplementary Figure 6. In breast TRN, we found several TFs had high betweenness centralities, suggesting that this network could be divided into multiple modules (Supplementary Figure 3 and 6A). However, TFs in blood and saliva TRNs had low betweenness centralities (Supplementary Figure 6B and 6C). We listed TFs ≥ 100 degrees in these TRNs in Supplementary Table 4. There were 23 TFs in breast TRN and 8 TFs in blood TRN; no TF ≥ 100 degrees were found in saliva TRN. In breast TRN, we also found 5 TFs ≥ 200 degrees; these TFs were NR1H4, HNF4A, POU4F2, PPARG and ZNF528. In addition, 8 TFs ≥ 100 degrees in breast and blood TRNs still have no data; most of them are zinc finger proteins (ZNF528, ZNF479, DMRT2, ZNF583, TBX15, ATOH8, ZNF367 and YBX2).

### Regulation type of TF-target genes in enriched pathways

**Figure 5:**
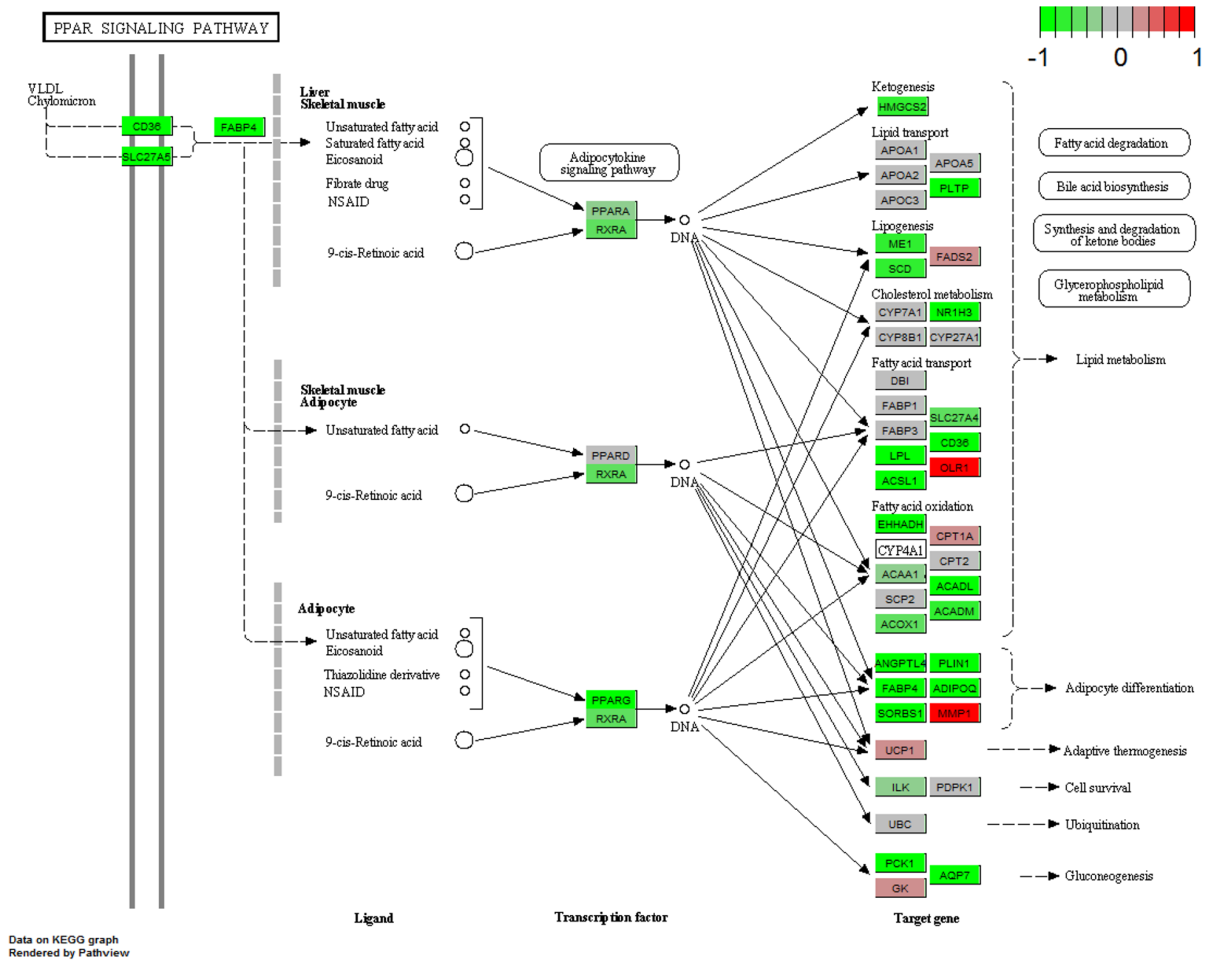
Gene expression profiles of the PPAR signaling pathway in breast tissue The red and green colors represent the log2(FC) of the corresponding genes.

**Figure 6:**
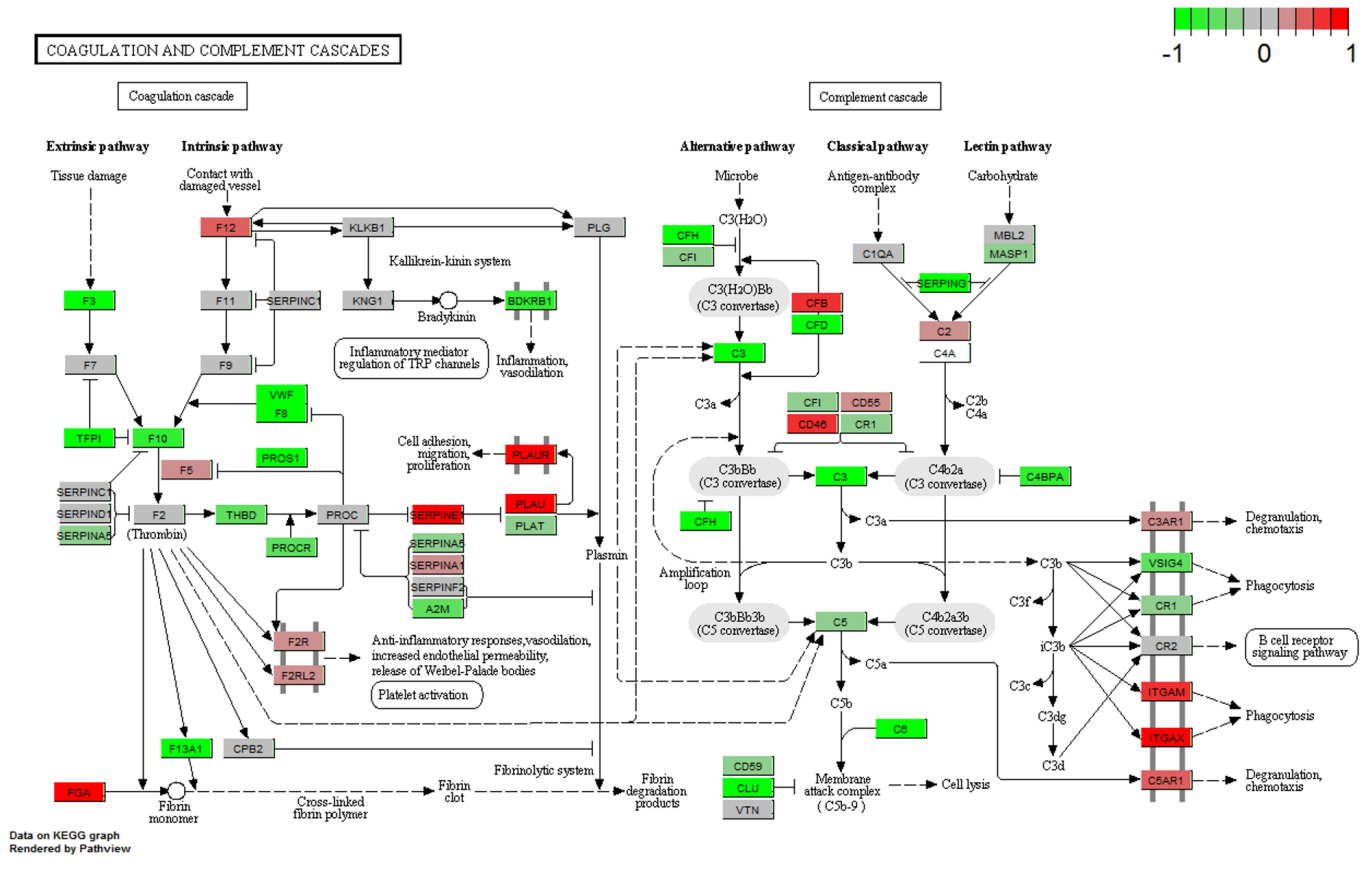
Gene expression profiles of the complement and coagulation cascades pathway in breast tissue The red and green colors represent the log2(FC) of the corresponding genes.

Numerous studies have demonstrated that the PPAR signaling pathway and complement and coagulation cascades are correlated with breast cancer pathology [16–21]. Therefore, we showed the regulation types and expression profiles of genes in these two pathways in breast (Figure 5 and 6). We also showed these pathways in the blood and saliva groups (Supplementary Figure 7-10). In Figure 5, the results showed that down-regulated PPARG suppresses many downstream genes in the PPAR signaling pathway. These genes were mainly involved in lipid metabolism, adipocyte differentiation, gluconeogenesis and other intracellular processes. In Figure 6, down-regulated F3 suppressed the expression of several downstream genes. Furthermore, up-regulated PALU and PALUR activated the cell adhesion, migration and proliferation functions. However, in the blood group, some genes in these two pathways showed opposite expression (Supplementary Figure 7 and 9). These two pathways were almost unaffected in the saliva group (Supplementary Figure 8 and 10).

## DISCUSSION

The present study showed a huge discrepancy in the global gene expression profiles, influenced pathways, transcription factor signatures and their transcriptional regulatory networks in breast, blood and saliva tissues in breast cancer. Among these three tissues, the most seriously impaired was the breast tissue. The blood tissue showed a relatively moderate damage, whereas the saliva tissue showed an almost undetectable difference.

Previous studies have identified numerous affected pathways and biological functions in breast cancer. Clarke et al. found a severe immune response dysfunction in breast cancer by a weighted gene coexpression network method [22]. A recent study identified several affected pathways such as cell cycle, cell adhesion and DNA replication in invasive ductal carcinoma (IDC), and the impairment pathways and dysregulated genes in IDC were different between the low-genetic-grade and high-genetic-grade groups [15]. Our previous study revealed a serious stromal genome heterogeneity between breast and prostate tumors and found that several metabolism- and cellular process-related pathways were affected in breast cancer, such as the tryptophan metabolism pathway and ABC transporters pathway [23]. In this study, we found 17 common up-regulated pathways (e.g., cell cycle, DNA replication, and p53 signaling pathway) both in breast and blood tissue. However, the 3 pathways showed contrary regulation between breast and blood. Only the ribosome pathway was up-regulated in the saliva group (Table 3). Furthermore, we performed GSEA using curated canonical pathways gene sets (http://software.broadinstitute.org/gsea/msigdb) to verify the above results. Our results showed that most of the enriched KEGG pathways are included in the enriched canonical pathways in both breast and blood tissues, no enriched canonical pathway in saliva (Supplementary Figure 11). These findings suggested that it had diversity in the impairment of pathways and biological functions in breast cancer for different tissues.

We identified 8 TFs with contrasting expression in breast and blood tissues based on differentially expressed gene analysis (CEBPD, EGR1, EGR2, EGR3, FOS, FOSB, ID1 and NFIL3). We also performed GSEA using transcription factor targets (TFT) gene set in three tissues. The gene sets contain genes that share a transcription factor binding site defined in the TRANSFAC (version 7.4, http://www.gene-regulation.com/) database. The identified TFs in breast and blood tissues are shown in Supplementary Table 5. However, we found only 17 dysregulated TFs in 90 TFT enriched TFs in breast tissue, and 2 up-regulated TFs in 22 TFT enriched TFs in blood tissue. FOSB is a member of the Fos gene family. These Fos genes encode leucine zipper proteins that can dimerize with proteins of the JUN family, thereby forming the transcription factor complex AP-1. The encoded FOS proteins have been shown to be involved in cell proliferation, differentiation, and transformation [24]. Early growth response proteins are a family of zinc finger transcription factors. The following are the four members of this family: EGR1, EGR2, EGR3 and EGR4. All of these TFs have been proven to correlate with breast cancer pathogenesis and prognosis [25–28]. In addition, this study found the following tissue specific TFs that were not reported in breast cancer: ATOH8, DMRT2, TBX15 and ZNF367. Down-regulated ATOH8 has been proven to contribute to the malignant phenotype of nasopharyngeal carcinoma [29] and increases the stem cell features of hepatocellular carcinoma cells [30]. Tun et al. reported that DMRT2 and other developmental TFs were significantly down-regulated in clear cell renal cell carcinoma [31]. Recently, genome-wide DNA methylation analysis suggested that TBX15 was hyper-methylated and down-expressed in hepatocellular carcinoma datasets [32]. However, contrary to these three TFs, ZNF367 was over-expressed in adrenocortical carcinoma, malignant pheochromocytoma, paraganglioma and thyroid cancer [33]. Interestingly, the expression patterns of these four TFs in our breast datasets were the same as those in previous reports. No expression change of these TFs was found in blood or saliva tissues.

The present study showed tissue-specific expressed TFs and target genes caused different impairment of biological functions in different tissues. We displayed these transcriptional regulation relationships in the PPAR signaling pathway and the complement and coagulation cascades pathway. EGR1 is necessary and sufficient to activate human peroxisome proliferator-activated receptor-γ1 (PPARG) gene expression, which has been verified in human aortic smooth muscle cells [34]. PPARG is a key regulator of lipogenic genes, and a previous mouse study demonstrated that PPARG plays a crucial role in hepatic lipid metabolism [35]. The present study showed that low-expressed EGR1 suppressed PPARG, and then low-expressed PPARG suppressed a series of downstream genes associated with lipogenesis, cholesterol metabolism, fatty acid transport and oxidation functions, thus resulting in abnormal lipid metabolism in breast tissue (Figure 3 and 5). Coagulation factor III, also known as tissue factor (F3, also known as TF), has been reported to be regulated by EGR1, is responsible for the initiation of the coagulation protease cascades by specific limited proteolysis [36, 37]. In breast tissue, down-regulated EGR1 suppressed F3 expression, caused the low-expression of several downstream genes and activated PLAU and PLAUR, eventually disturbing cell adhesion, proliferation and metastasis functions (Figure 5 and 6). Furthermore, low-expressed FOS suppressed CLU and inhibited cell lysis function in breast tissue. However, EGR1 and FOS were all up-regulated in blood tissue, and some downstream genes showed high-expression. No expression change was observed in saliva tissue (Figure 3, Supplementary Figure 7 and 8). It is worth noting that these functions were correlated with breast cancer pathological processes, and the extent of damage in these pathways varied largely in different tissues. Our study also found a series of unreported tissue-specific TFs that may correlate with breast cancer. However, no TF-target data were provided. Therefore, future studies need to verify these correlations.

In conclusion, our study identified a series of tissue-specific TFs that correlated with breast cancer. Some of them are novel, such as ATOH8, DMRT2, TBX15 and ZNF367. These TFs may be used as biomarkers for accurate diagnosis and prognosis or as predictive markers for treatment efficiency. Furthermore, we found these dysregulated TFs and their target genes impaired lipid metabolism, the coagulation cascade, cell adhesion, proliferation, differentiation and metastasis processes. The extent of damage in these functions varied widely in breast, blood and saliva tissues. These results suggest that the tissue-specific gene expression in breast cancer would require careful consideration in future clinical practice and theoretical research.

## MATERIALS AND METHODS

### Microarray data collection and preprocessing

Human breast cancer microarray datasets were searched and downloaded from the NCBI-GEO database ( http://www.ncbi.nlm.nih.gov/geo) in March 2016. We used the keywords of “breast cancer”, “breast adenocarcinoma” and “breast tumor” to perform accurate searching. The data selection criteria were as follows: (1) all datasets were genome-wide; (2) the samples of each data set must include breast cancer patients and controls; (3) the number of cases and controls in each dataset must be ≥ 3; (4) all samples were non-cell-line samples; and (5) complete microarray raw or normalized data were available. Based on the above criteria, we have finally chosen 14 datasets for our integrated analysis (GSE8977, GSE10810, GSE16391, GSE20266, GSE26910, GSE27562, GSE29431, GSE31192, GSE35925, GSE36765, GSE42568, GSE45827, GSE50567, and GSE61304). The integrated datasets included 621 breast cancer patients and 208 controls. Details of all datasets could be seen in Table 1. All the datasets were tested using the platform of Affymetrix Human Genome U133 Plus 2.0 Array. Among them, 11 datasets were tested using breast tissue (including 470 patients and 163 controls), 2 datasets were tested using blood (including 141 patients 35 controls) and 1 dataset was tested using saliva (including 10 patients and 10 controls). Thus, we divided these datasets into 3 subgroups based on the sample collection source including breast, blood and saliva.

R v3.2.2 was used to perform data preprocessing. We used the Robust Multichip Average (RMA) algorithm in oligo package [38] to normalize the raw expression data and generate normalized gene expression intensity. Gene annotation, integration and renormalization of the 14 datasets were carried out using a custom written Python code. We have removed probes with no gene annotation or that matched multiple gene symbols. Next, we calculated the average expression value of multiple probe IDs that matched to an official gene symbol and took this value to represent the expression intensity of the corresponding gene symbol. The renormalization method and scripts are described in our previous publications [39, 40]. The distributions of RMA processed and global renormalized gene expression values across all studies are shown in Supplementary Figure 1 and 2. After the global expression was renormalized, the distribution of gene expression values across all studies had a consistent range.

### Differential expression genes analysis

Differential expression gene analysis was performed using R v3.2.2 and the Bioconductor Library. The empirical Bayes algorithm (function “eBayes”) in the limma package [41] was used to detect differentially expressed genes between breast cancer patients and controls. Significantly up-regulated genes were defined by as a logarithmic transformed fold-change (log2(FC)) ≥ log2(1.5) and a false discovery rate (FDR) adjusted P value ≤ 0.05. Significantly down-regulated genes were defined by a log2(FC) ≤ -log2(1.5) and an FDR-P value ≤ 0.05. We carried out the differential expression analysis in three tissues.

### Gene set enrichment analysis

We used javaGSEA Desktop Application v2.2.2 to perform gene set enrichment analysis (GSEA) of breast cancer datasets. We chose KEGG pathway enrichment analysis to compare the impaired pathways in breast, blood and saliva tissues and tried to find the correlations between TFs and impaired pathways. The curated KEGG gene sets v5.1 (including 186 gene sets) ( http://software.broadinstitute.org/gsea/msigdb/genesets.jsp?collection=CP:KEGG) were chosen to perform KEGG pathway enrichment analysis among the three groups. Additionally, the gene sets less than 15 genes or more than 500 genes were excluded. The phenotype label was set as breast cancer vs. control. The t-statistic mean of the genes was computed in each KEGG pathway using a permutation test with 1000 replications. The up-regulated pathways were defined by a normalized enrichment score (NES) > 0 and the down-regulated pathways were defined by an NES < 0. Pathways with an FDR-P value ≤ 0.05 were chosen as significantly enriched. We used Venn diagram in InteractiVenn ( http://www.interactivenn.net/) [42] to show the enriched KEGG pathways among these groups.

### Transcription factor analysis

We downloaded 1,544 human transcription factors (TFs) from the Animal Transcription Factor Database (AnimalTFDB, http://www.bioguo.org/AnimalTFDB/index.php) [43] and mapped 1,469 TFs to our integrated datasets. We filtered TFs that were differentially expressed in two and more groups and used the TRRUST web server ( http://www.grnpedia.org/trrust/) [44] to find the target genes of the commonly dysregulated TFs. TRRUST could provide the information of the regulation type (such as activation and repression) between the queried TFs and target genes. We used heatmap in the “pheatmap” package to show the expression profiles of TFs and TF-target genes in the three groups.

Reconstruction of tissue-specific transcriptional regulatory networks (including breast, blood and saliva) were used GENIE3 software [45]. We used the gene expression matrix of the three tissues and transcriptional regulation relationship list in TRRUST as the input data and ran GENIE3 with its default parameters. The original output contained 4.12E8 TF-target interactions and we extracted the top 10,000 interactions. Next, we used Cytoscape v3.2.1 to visualize the output results. We used the NetworkAnalyzer tool in Cytoscape to perform network analysis of the three networks.

Based on previous reports, the PPAR signaling pathway and complement and coagulation cascades were critical in breast cancer pathology [16–21]. Therefore, we chose these two pathways and showed the expression profiles of the corresponding genes. We used the “pathview” package [46] to display these results. This package could provide the links between genes and pathways based on the KEGG pathway. We showed the gene expression profiles, their interactions and regulations, and related functions in the selected pathways in each group (breast, blood and saliva).

## SUPPLEMENTARY MATERIALS FIGURES AND TABLES


