# Supplementary material for: Comprehensive tissue-specific gene set enrichment analysis and transcription factor analysis of breast cancer by integrating 14 gene expression datasets
Source: Oncotarget. 2016 Dec 21;8(4):6775–86. doi: 10.18632/oncotarget.14286 (PMC5351668; doi:10.18632/oncotarget.14286)
Supplement: Supplementary file 1 [file oncotarget-08-6775-s001.pdf]

Comprehensive tissue-specific gene set enrichment analysis and transcription factor analysis of breast cancer by integrating 14 gene expression datasets

SUPPLEMENTARY FIGURES AND TABLES

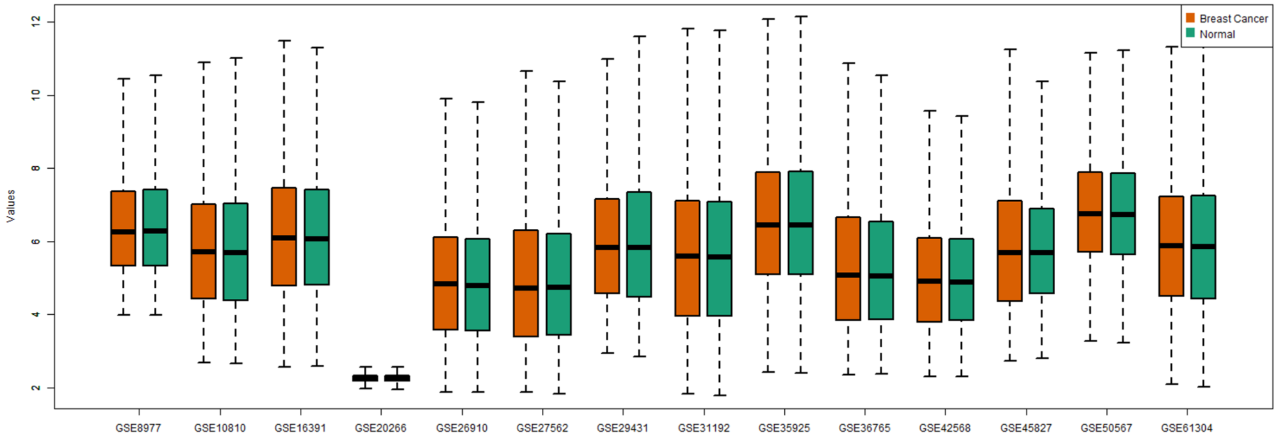

Supplementary Figure 1: The distribution of RMA processed gene expression values of breast cancer datasets. The orange box showed the distribution of gene expression values in breast cancer patients and the green box showed the distribution of gene expression values in controls. There was a relatively large deviation in the distribution of gene expression values across these studies.

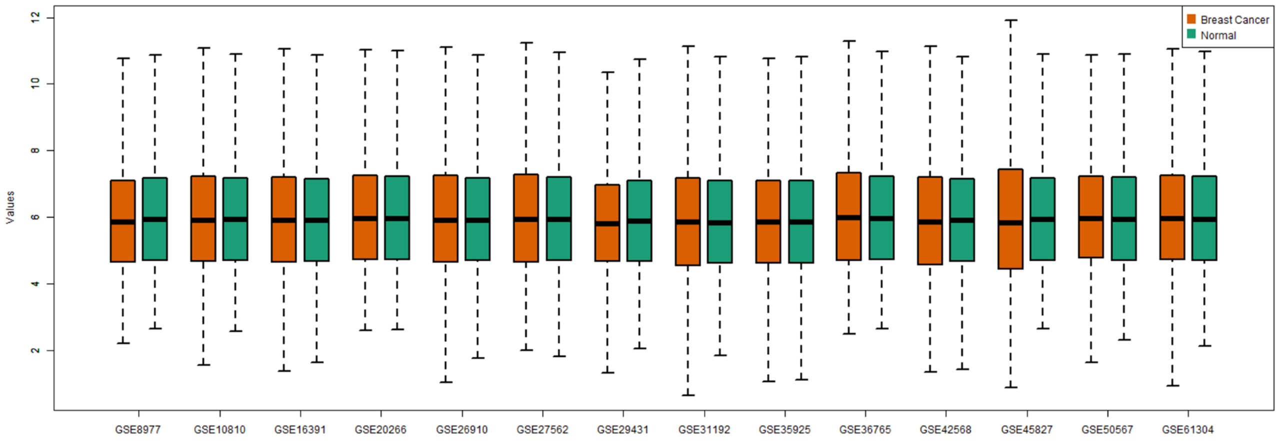

Supplementary Figure 2: The distribution of global renormalized (after RMA processed) gene expression values of breast cancer datasets. The orange box showed the distribution of gene expression values in breast cancer patients and the green box showed the distribution of gene expression values in controls. The distribution of gene expression values across these studies had a consistent range.

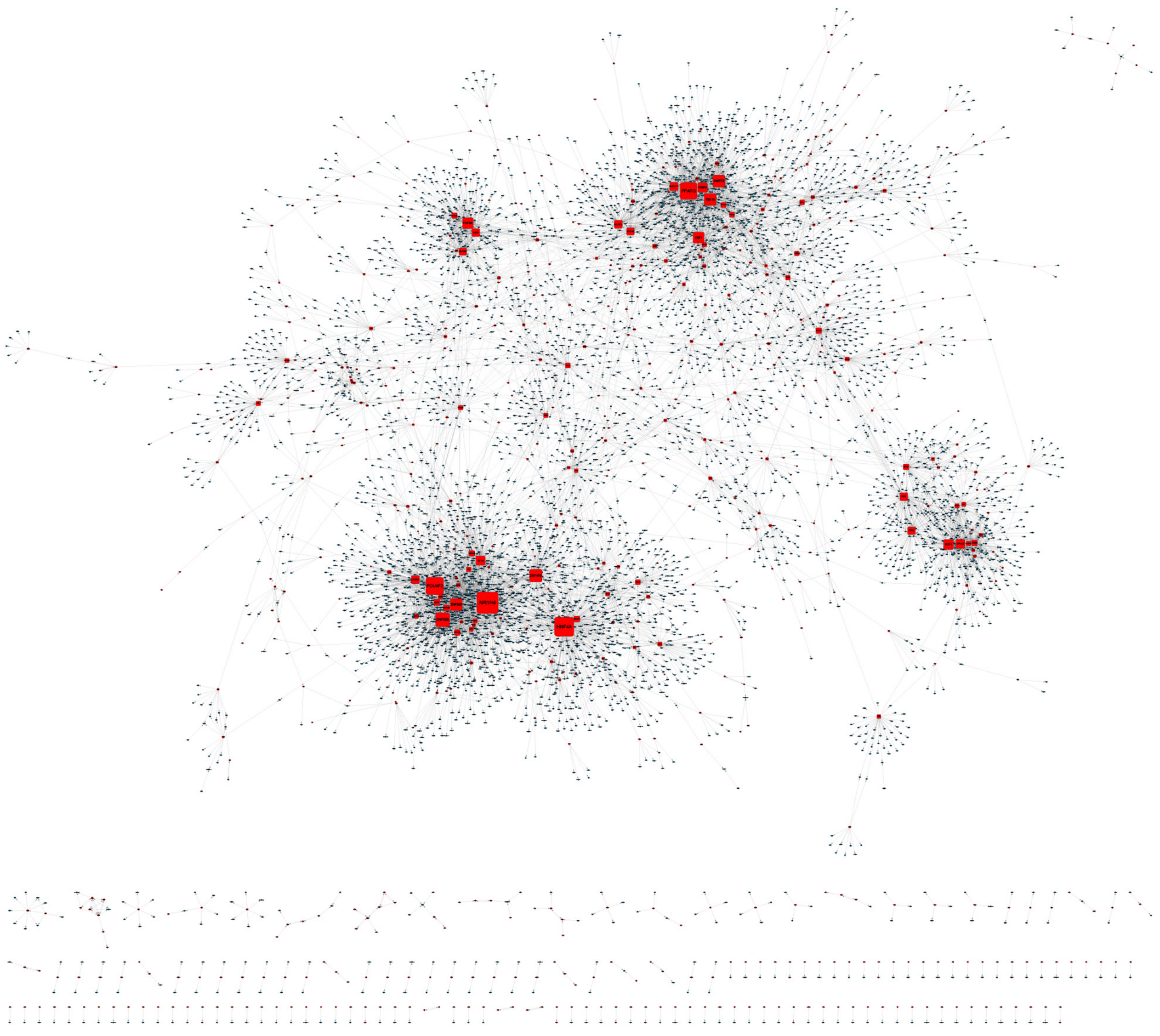

Supplementary Figure 3: Transcriptional regulatory networks of breast tissus. The red boxes represent the TFs and blue boxes represent the target genes. Box size represents the number of nodes of a gene. Arrows indicate the direction of regulation.

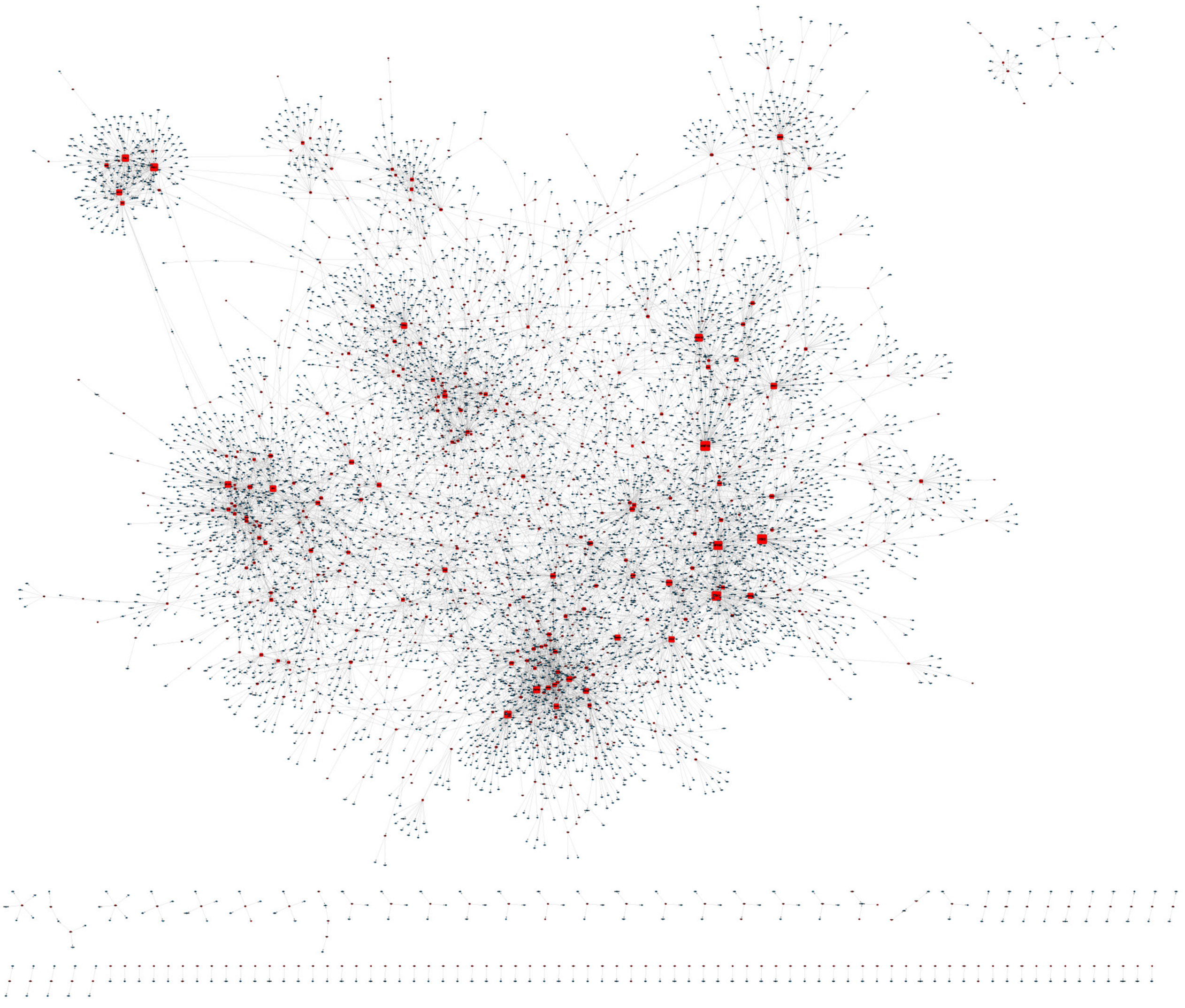

Supplementary Figure 4: Transcriptional regulatory networks of blood tissus. The red boxes represent the TFs and blue boxes represent the target genes. Box size represents the number of nodes of a gene. Arrows indicate the direction of regulation.

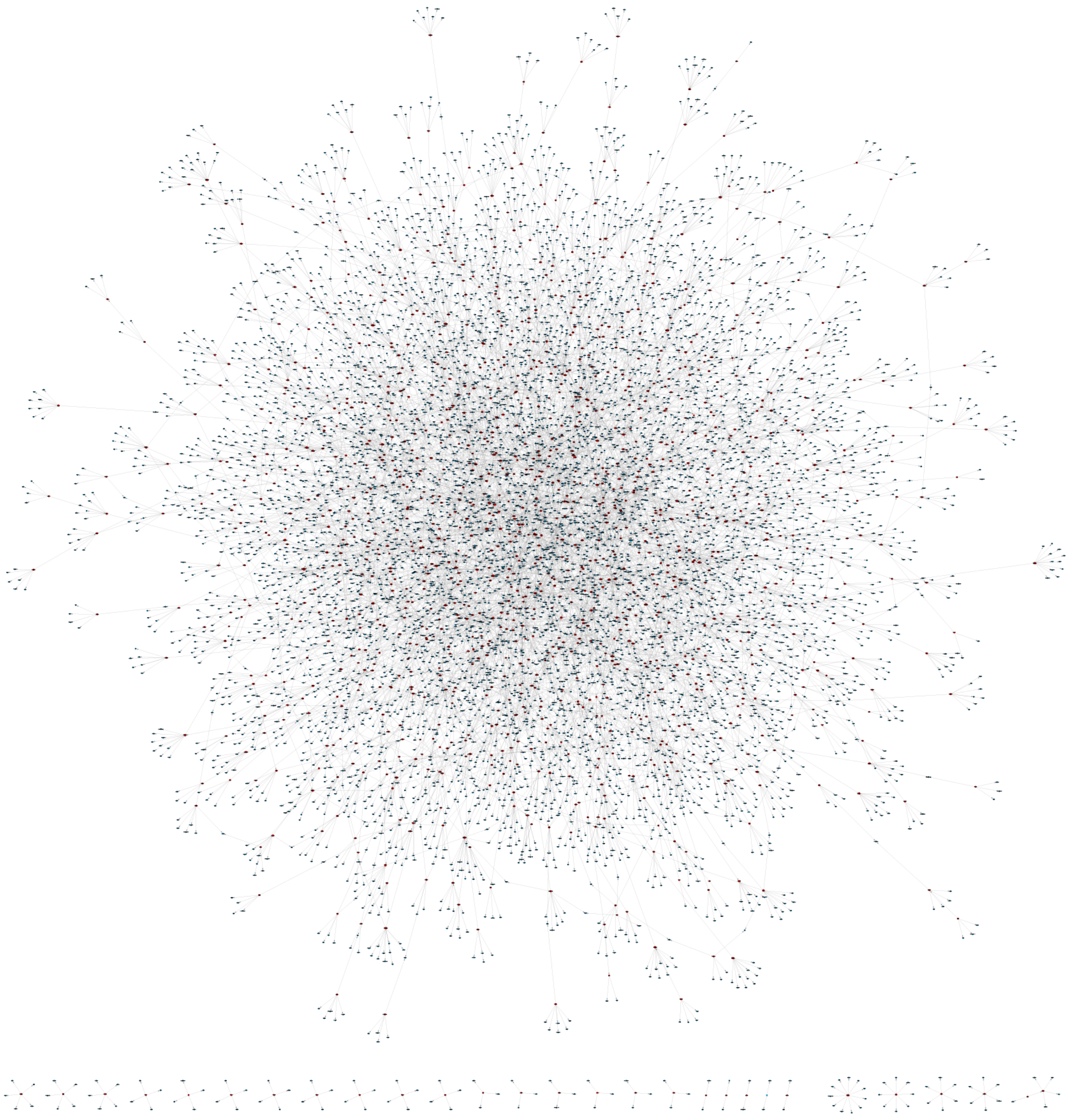

Supplementary Figure 5: Transcriptional regulatory networks of saliva tissus. The red boxes represent the TFs and blue boxes represent the target genes. Box size represents the number of nodes of a gene. Arrows indicate the direction of regulation.

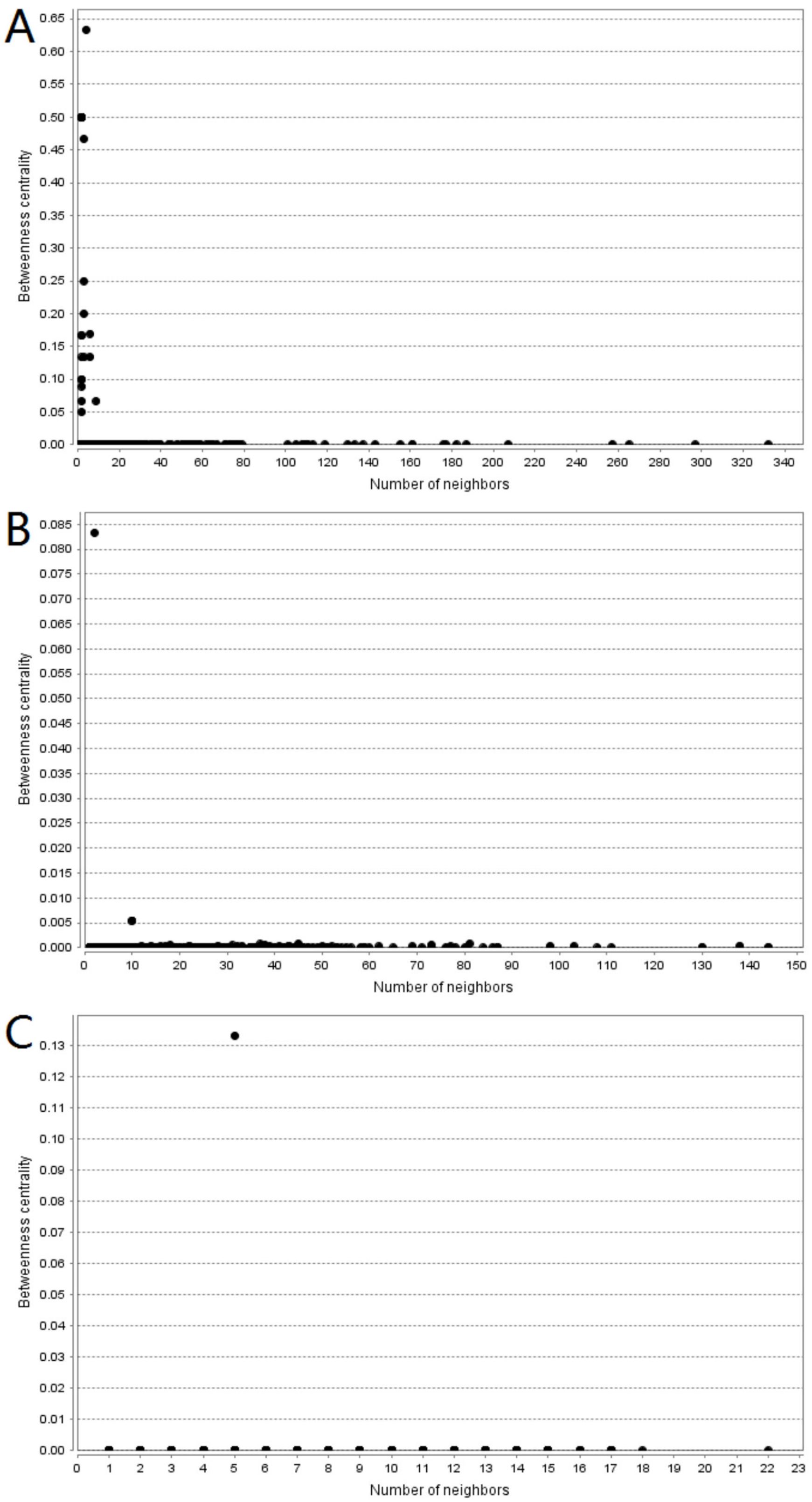

Supplementary Figure 6: Betweenness centrality of breast A. blood B. and saliva C. transcriptional regulatory networks.

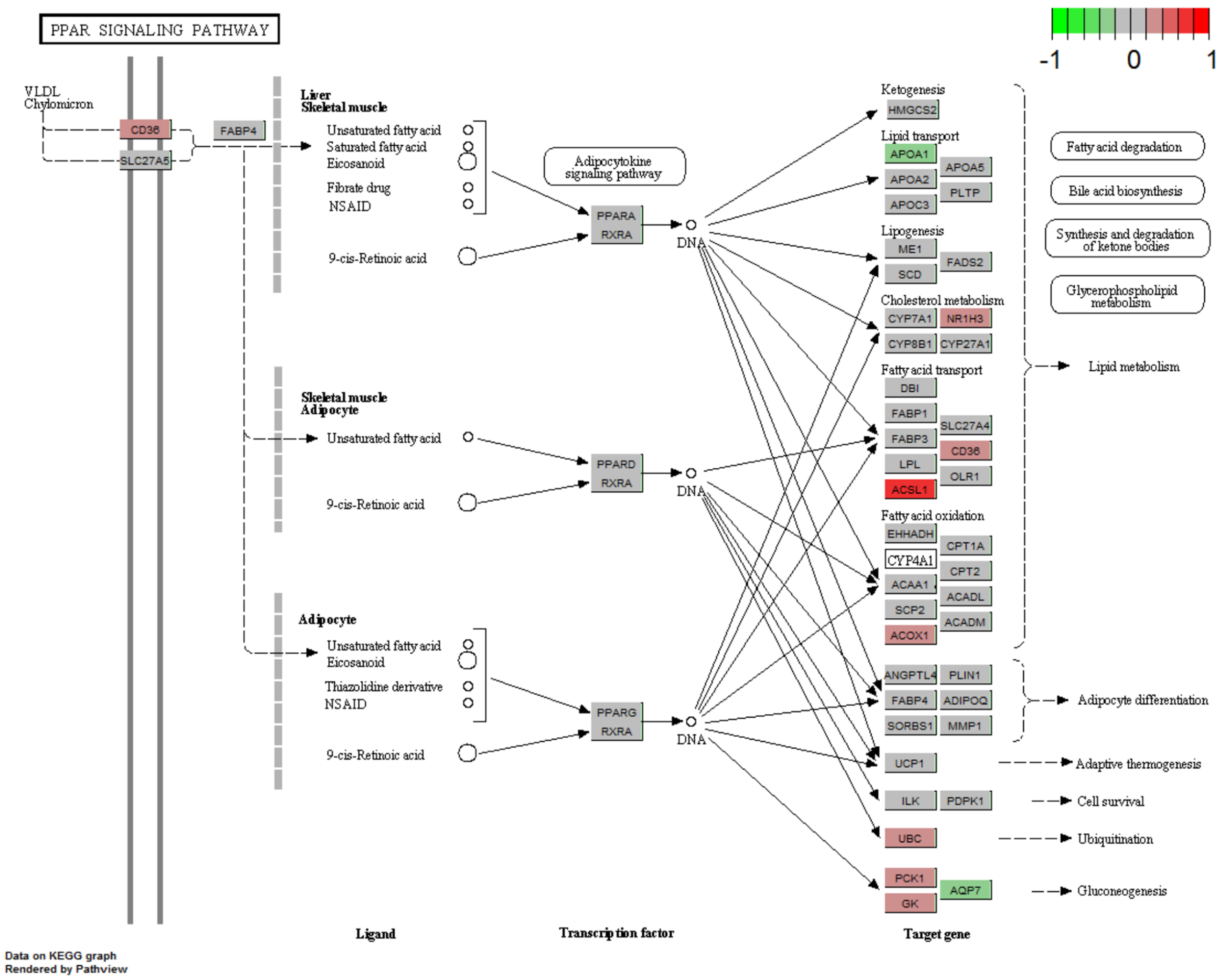

Supplementary Figure 7: Gene expression profiles of PPAR signaling pathway in blood. The red and green color represented the log2(FC) of the corresponding genes.

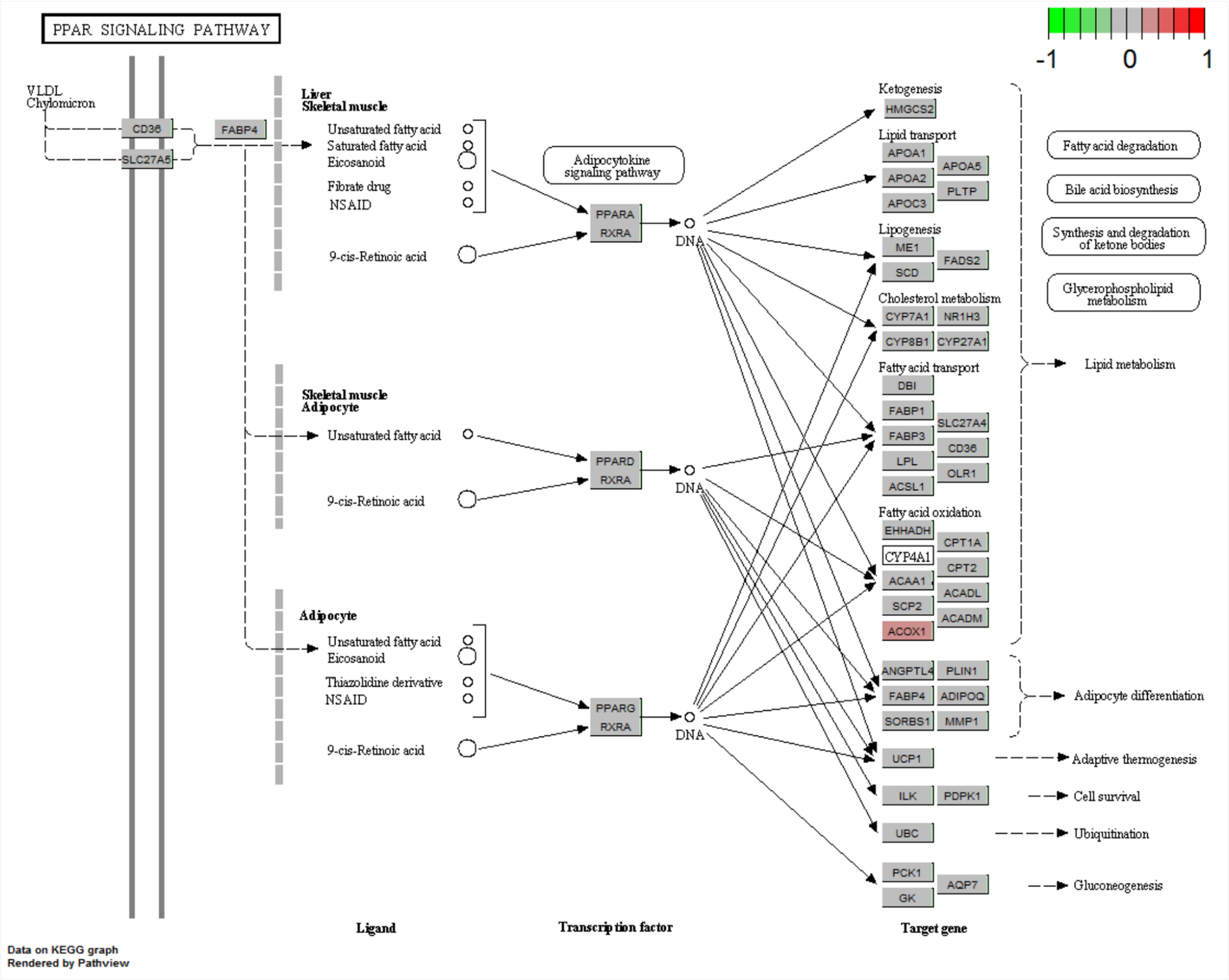

Supplementary Figure 8: Gene expression profiles of PPAR signaling pathway in saliva. The red and green color represented the log2(FC) of the corresponding genes.

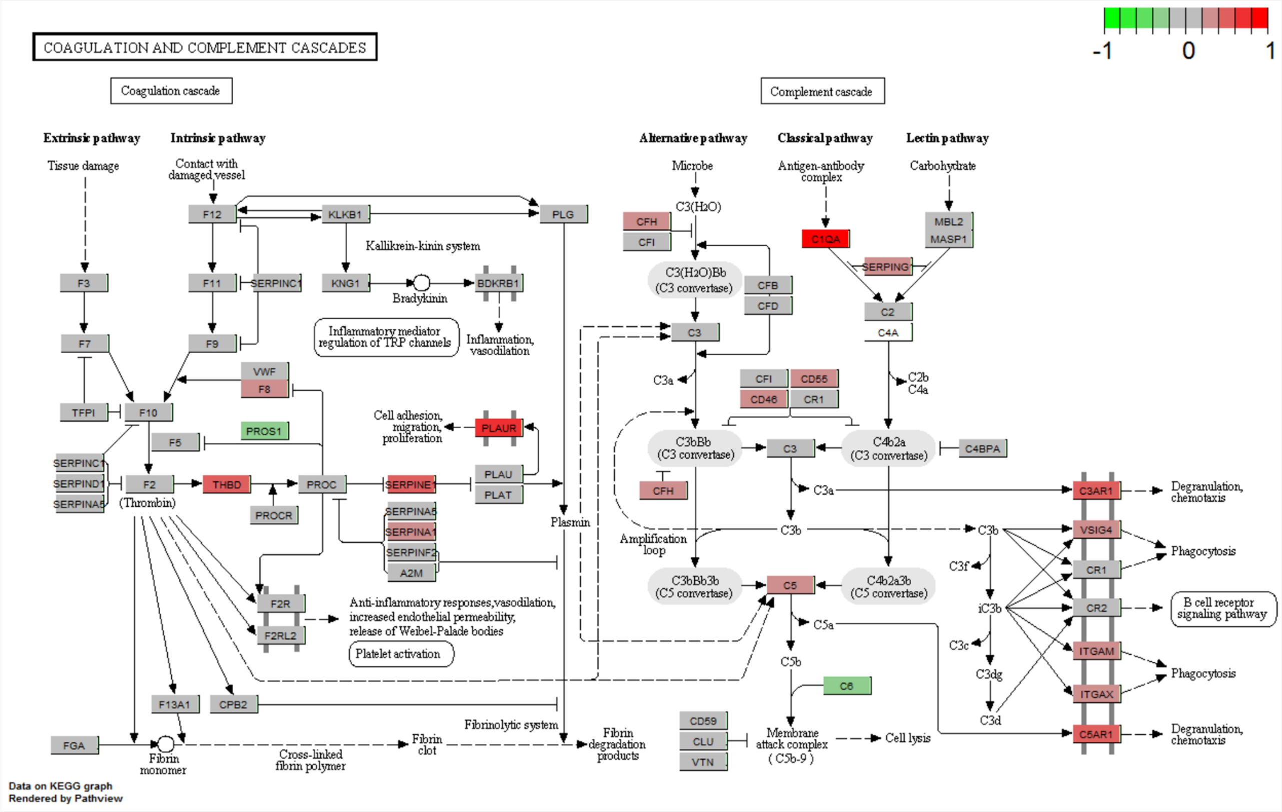

Supplementary Figure 9: Gene expression profiles of complement and coagulation cascades pathway in blood. The red and green color represented the log2(FC) of the corresponding genes.

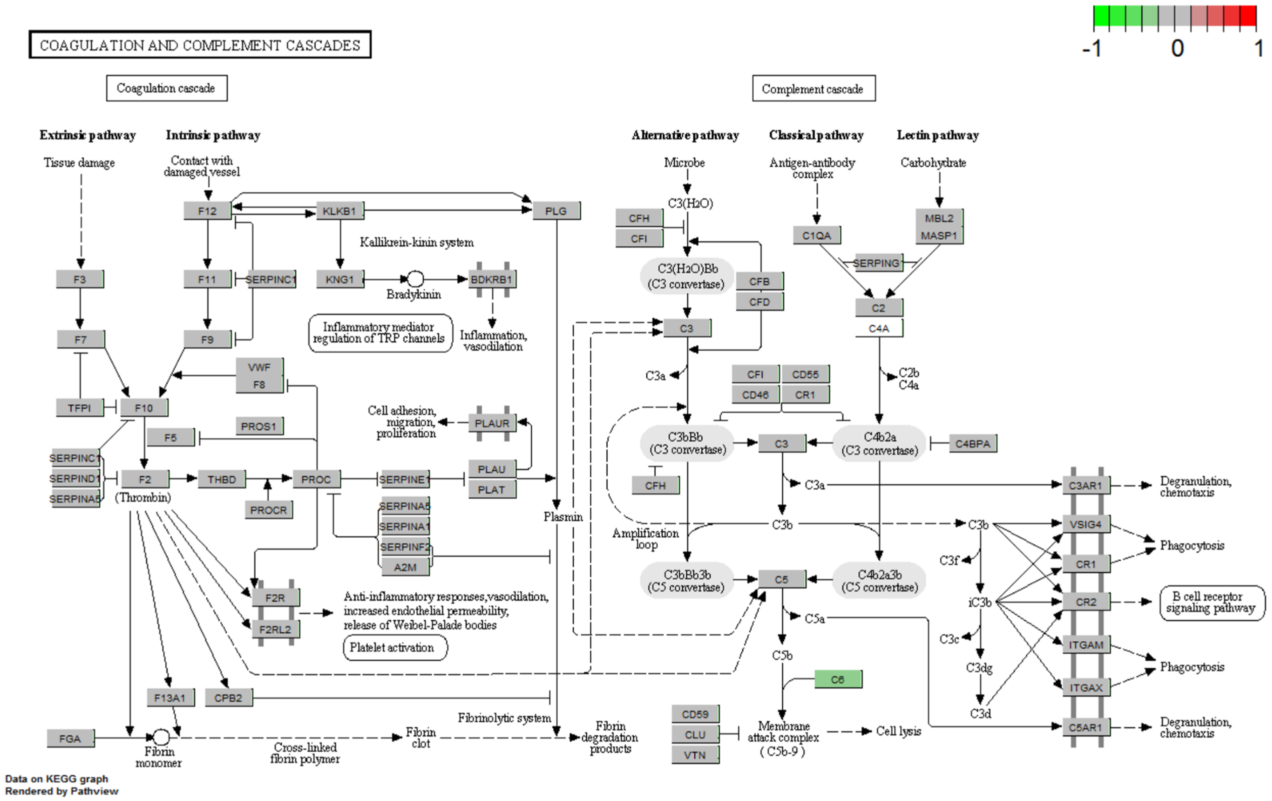

Supplementary Figure 10: Gene expression profiles of complement and coagulation cascades pathway in saliva. The red and green color represented the log2(FC) of the corresponding genes.

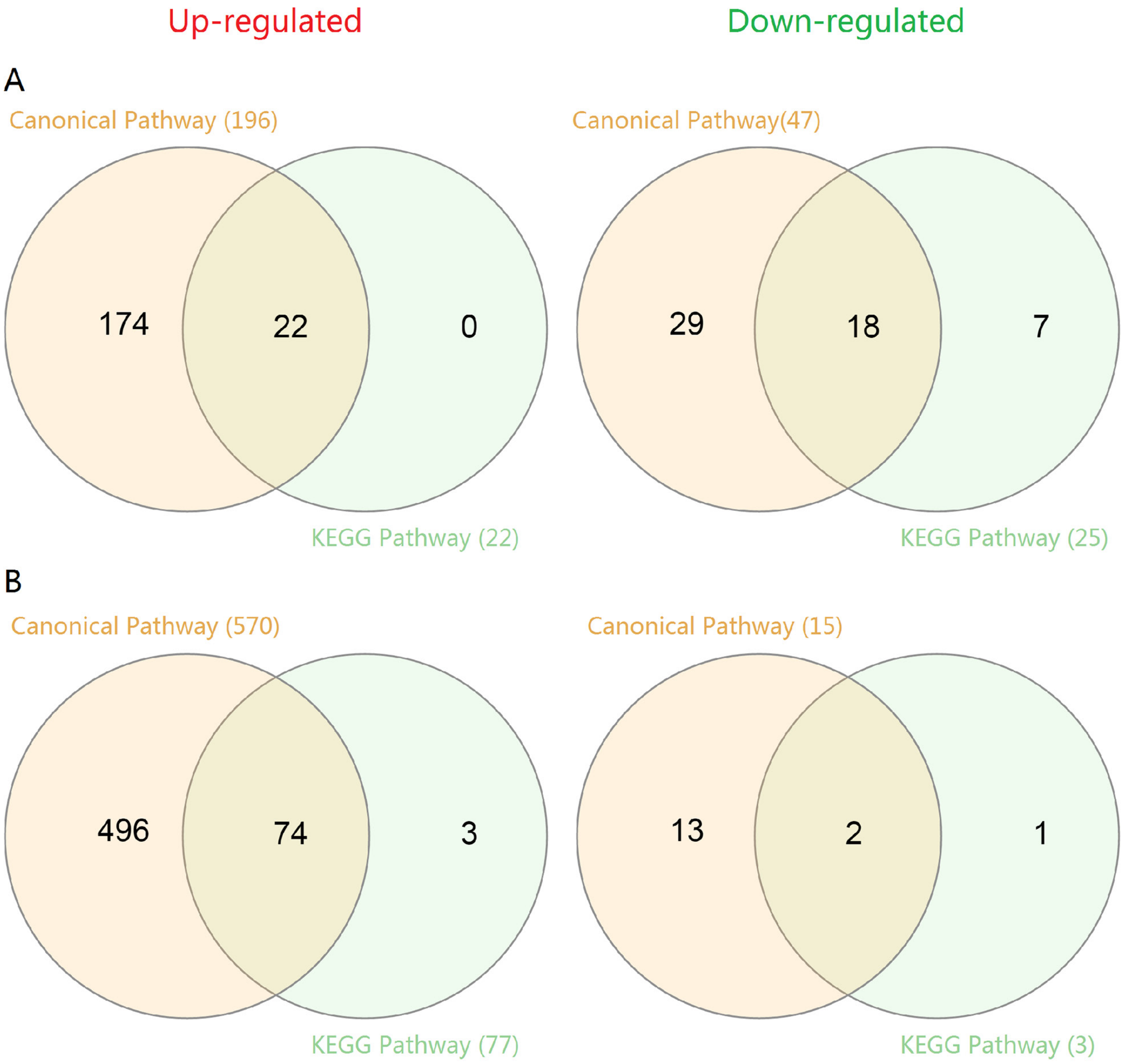

Supplementary Figure 11: Venn diagram of enriched canonical pathways and KEGG pathways by GSEA method. Panel **A.** showed the up- and down-regulated pathways in the breast tissue. Panel **B.** showed the up- and down-regulated pathways in the blood tissue.

Supplementary Table 1: Commonly and specific dysregulated genes in breast and blood[1]

Commonly up-regulated	Commonly down-regulated	Up-regulated in breast and down-regulated in blood	Down-regulated in breast and up-regulated in blood
ARF4	EPB42	CENPK	ALDH1A1
BAX	HBD	SNORD104	CEBPD[2]
CXCR4			EGR1[2]
DUSP5			EGR2[2]
IER3			EGR3[2]
IL8			EPS8
LMNB1			FOS[2]
NCEH1			FOSB[2]
PLAUR			G0S2
PRC1			ID1[2]
PRDX1			NFIL3[2]
RGS1			PFKFB3
RRM2			PTGS2
THOC4			PTX3
TMEM49			ZFP36
UBE2M			

[1] Novel finding genes were colored in red.
[2] This gene is a transcription factor.

Supplementary Table 2: Dysregulated transcription factors in breast and blood[1]

Breast						Blood	
Up			Down			Up	Down
ARNT2	LASS2	ZNF217	ARID5A	ID1	RFX6	CEBPB	
BHLHE40	LASS6	ZNF239	ATOH8	ID4	RHOXF1	CEBPD	
CREB3L4	LEF1	ZNF267	BCL6	IRX1	SATB1	EGR1	
DNAJC1	MYB	ZNF281	CEBPA	JUN	SIM1	EGR2	
E2F3	MYBL1	ZNF322B	CEBPD	KLF10	SMAD9	EGR3	
E2F5	MYBL2	ZNF367	CREB5	KLF15	SOX10	FOS	
E2F7	NFE2L3	ZNF468	DMRT2	KLF2	SOX17	FOSB	
E2F8	OVOL1	ZNF587	EBF1	KLF4	SOX7	ID1	
ELF3	PITX1	ZNF623	EBF3	KLF9	STAT5A	IRF2	
ELF4	RFX5	ZSCAN16	EGR1	MAF	TBX15	JUNB	
EN1	SIX4		EGR2	MAFF	TCF4	MAFB	
FOXA1	SMARCC1		EGR3	MEIS2	TCF7L1	NFIL3	
FOXM1	SOX11		ELF5	MEOX1	TCF7L2	NR4A2	
FOXO6	SOX4		EMX2	MEOX2	THRB		
GATA3	SOX9		ERG	MLXIPL	TWIST1		
GRHL2	STAT1		FOS	MSX1	TWIST2		
GTF2I	TEAD4		FOSB	MXI1	ZBED3		
HES6	TFAP2A		FOXC1	NFIA	ZBTB16		
HMGB3	TFEC		FOXO1	NFIB	ZBTB20		
HOXC10	TGIF1		HEY1	NFIL3	ZBTB4		
HOXC13	TOX3		HLF	NR1H3	ZEB2		
INSM1	TRPS1		HOXA10	NR2F1	ZFPM2		
IRF6	ZBTB41		HOXA3	NR3C1	ZNF423		
IRF7	ZBTB42		HOXA4	NR3C2	ZNF502		
IRF9	ZIC2		HOXA5	OSR1	ZNF521		
IRX5	ZNF146		HOXA7	PLAGL1	ZNF559		
KDM5B	ZNF165		HOXD8	PPARG	ZNF662		

[1] Novel finding transcription factors were colored in red.

Supplementary Table 3: Transcriptional regulatory network properties of three tissues

Parameters	Breast	Blood	Saliva
Clustering coefficient	0.083	0.038	0.000
Connected component	153	116	28
Characteristic path length	4.917	6.081	1.779
Ave. number of neighbors	3.349	2.984	2.370
Number of nodes	5915	6665	8433
Multi-edge node pairs	95	55	9

Supplementary Table 4: Selected transcription factors in breast and blood transcriptional regulatory networks[1]

Breast		Blood	
**Gene symbol**	**Degree[2]**	**Gene symbol**	**Degree[2]**
NR1H4	335	HNF1B	144
HNF4A	297	YBX2	144
POU4F2	267	ZHX1	138
PPARG	258	SPDEF	130
ZNF528	211	PBX1	112
ZNF479	187	PKNOX2	108
DMRT2	182	NFYB	105
ZNF583	179	TAL1	100
TBX15	178		
EBF1	163		
FOXM1	158		
IKZF1	145		
CEBPA	139		
ST18	134		
SP140	132		
RHOXF1	119		
CREB1	113		
MLXIPL	113		
IRF8	111		
E2F8	111		
TFEC	109		
ATOH8	106		
ZNF367	102		

[1] Novel finding transcription factors were colored in red.
[2] TFs with a degree ≥ 100 were selected.

Supplementary Table 5: Screened transcription factors by GSEA using transcription factor targets (TFT) gene set (no enriched gene set in saliva)

Breast				Blood
ALX4	FOXJ1	MYOG	SOX5	AHR
AR	FOXJ2	NFE2	SOX9[1]	ARNT
ATF2	FOXM1[1]	NFE2L1	SRF	ATF2
CDX2	FOXQ1	NFIL3[1]	SRY	CEBPA
CEBPA[1]	GABPA	NKX2-2	STAT1[1]	CEBPB[1]
CEBPB	GABPB1	NKX3-1	STAT4	CREB1
CEBPD[1]	GATA1	NKX6-1	STAT5A[1]	E2F1
CEBPG	GATA3[1]	NKX6-2	STAT5B	ELK1
CRX	GATA4	NR3C1[1]	TAL1	ETS1
DBP	GATA6	NRF1	TBP	ETV7
E2F1	GFI1	PAX2	TCF3	GABPA
E2F4	GTF2A1	PAX4	TEAD1	GABPB1
ELK1	HAND1	PAX6	TLX2	JUN
EN1[1]	HLF[1]	PBX1	VSX1	MAX
ESR1	HNF4A	PGR	YY1	MYC
ETS2	HOXA4[1]	PITX2	ZHX2	NFIL3[1]
ETV7	HOXA5[1]	POU2F1	ZIC3	NRF1
FOXA1[1]	HSF2	POU3F1	ZNF238	PAX3
FOXA2	JUN[1]	POU3F2		REST
FOXC1[1]	LHX3	POU6F1		SREBF1
FOXD1	LMO2	PPARA		SRF
FOXD3	MEF2A	PRRX2		YY1
FOXF2	MYB[1]	RFX1		
FOXI1	MYC	RORA		

[1] This transcription factor was differentially expressed.
